# ZnO nanowires array grown on Ga-doped ZnO single crystal for dye-sensitized solar cells

**DOI:** 10.1038/srep11499

**Published:** 2015-06-23

**Authors:** Qichang Hu, Yafeng Li, Feng Huang, Zhaojun Zhang, Kai Ding, Mingdeng Wei, Zhang Lin

**Affiliations:** 1 Key Laboratory of Optoelectronic Materials Chemistry and Physics, Fujian Institute of Research on the Structure of Matter, Chinese Academy of Sciences, Fuzhou, Fujian, 350002, People’s Republic of China; 2 State Key Laboratory of Photocatalysis on Energy and Environment, Fuzhou University, Fuzhou, Fujian, 350002, People’s Republic of China; 3 State Key Laboratory of Optoelectronic Materials and Technologies, School of Physics and Engineering, Sun Yat-Sen University, Guangzhou, Guangdong, 510275, People’s Republic of China

## Abstract

High quality ZnO nanowires arrays were homoepitaxial grown on Ga-doped ZnO single crystal (GZOSC), which have the advantages of high conductivity, high carrier mobility and high thermal stability. When it was employed as a photoanode in the DSSCs, the cell exhibited a 1.44% power-conversion efficiency under the illumination of one sun (AM 1.5G). The performance is superior to our ZnO nanowires/FTO based DSSCs under the same condition. This enhanced performance is mainly attributed to the perfect interface between the ZnO nanowires and the GZOSC substrate that contributes to lower carrier scattering and recombination rates compared with that grown on traditional FTO substrate.

Nowadays, fossil energy shortage and environmental pollution push people to focus on renewable solar energy utilization. Dye-sensitized solar cells (DSSCs), one of the representative photovoltaic devices, have attracted extensive attention due to their low cost, simple fabrication and high theoretical power conversion efficiency (PCE)^1^,^2^,^3^,^4^,^5^,^6^. Although porous TiO_2_ nanocrystalline film is widely used as the photoanode material of DSSCs, ZnO is regarded as a potential alternative to TiO_2,_ due to its easy crystallization and anisotropy growth, which makes it much easier for ZnO to form nanowire arrays^7^,^8^,^9^. Compared with nanocrystalline network TiO_2_, ZnO nanowire arrays generally have higher crystallinity and fewer grain boundaries, which could reduce grain-boundary scattering and electrons back reaction with the surrounding electrolyte^9^,^10^,^11^. Theoretically, high-quality ZnO nanowire arrays would greatly promote the conversion efficiency of DSSCs.

However, traditional ZnO nanowires arrays used in photoanode are mostly grown on conductive glass^10^,^11^,^12^,^13^,^14^,^15^,^16^,^17^, such as FTO, ITO, AZO films, etc. The crystallinity of those polycrystalline films is far worse than that of single crystal. There exist many defects at the interface of the substrate and the nanowires due to lattice and thermal mismatch, which will consequently reduce the crystallinity of and electrical property of ZnO^17^,^18^,^19^. And the photoanode prepared on a polycrystalline film will lead to lower mobility, much lower chemical and thermal stability. That’s why the efficiency of nanowire arrays DSSCs has still not been greatly improved.

Apparently, homoepitaxial growth is the best option to solve the above problems. Single crystal Ga-doped Zn (GZOSC) has the same crystal parameter as pure ZnO, but it has much higher conductivity, thus it is an ideal substrate for growing the ZnO nanowires used in DSSCs^20^,^21^,^22^,^23^. It is expected that high-quality homogeneous interface between the GZOSC and the nanowires could be obtained, and consequently avoiding the scattering and recombination of electrons at the interface, as shown in the right of Fig. 1. The instability of the device caused from the instability of polycrystalline ZnO can also be improved. As far as we are concerned, the oriented ZnO nanowires arrays grown on GZOSC has never been employed in the field of DSSCs.

In this work, a novel ZnO nanowire arrays based DSSCs, whose photoanode is prepared by homoepitaxial growth on GZOSC, is reported for the first time. It consists of GZOSC, ZnO nanowire, dye sensitizer, [I^−^/I_3_^−^] electrolyte and Pt counter electrode, in which the traditional conductive glass has been replaced (see the left part of Fig.1). The performance of GZOSC based DSSCs was found to be superior to FTO based DSSCs under the same growing condition. Therefore, GZOSC based photoanode has great potential to improve the performance of DSSCs.

The features of the device are as follows: Firstly, the GZOSC used in DSSCs is grown by the independent research of our group^20^,^21^,^22^, with high carrier mobility and carrier concentration, as well as high thermal and chemical stability. The application of such material with high conductivity to cell is beneficial to reduce its internal resistance and to improve its electron transmission efficiency^24^. And the high thermal and chemical stability of GZOSC is expected to greatly improve the stability of ZnO-based DSSCs. So our work has certain realistic significance.

Secondly, the single crystal orientation of ZnO nanowires is achieved when the single crystal surface serves as the substrate. It is contributed to get reliable results when studying the relationship between charge transferring and the crystal plane. So our work has specific scientific research value.

## Results

**Figure 2:**
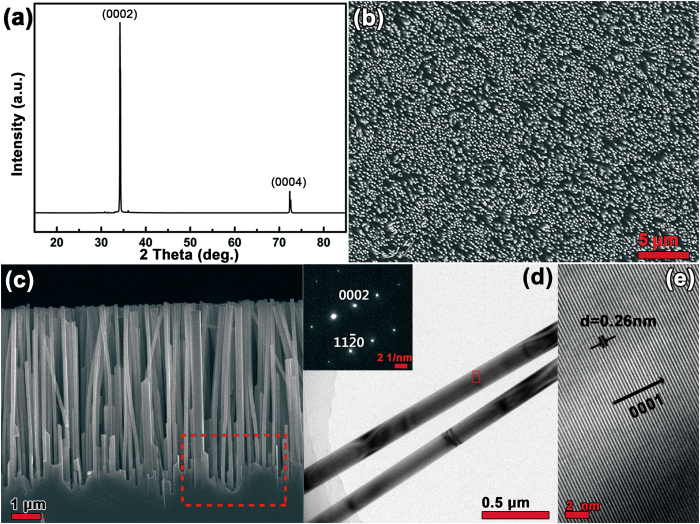
(**a**) XRD spectrum of the ZnO nanowire array/GZOSC photoanode. (**b**) Top view SEM image of the photoanode. (**c**) Cross-sectional SEM images of the photoanode. (**d**) Low-resolution TEM image of two individual ZnO nanowire in the photoanode and the upper left inset gives its corresponding SAED pattern. (**e**) HRTEM image taken from the area marked with the red frame in (**d**).

Figure 2a shows the XRD pattern of the ZnO nanowire/GZOSC photoanode. Two sharp narrow peaks at 34.4° and 72.4° in Fig. 2a can be indexed to a single crystalline wurtzite structure of ZnO. The dominated (002) peaks indicate an upstanding ZnO nanowire arrays along the c-axis. The XRD patterns of bare GZOSC and ZnO nanowire/GZOSC shown in the Figure S1 indicate that GZOSC has almost the same lattice parameter as pure ZnO. Top view SEM image of ZnO nanowire is shown in Fig. 2b. It can be seen that the top surface is uniform and orderly, indicating the nanowire is almost perpendicular to the substrate. Cross-sectional SEM image of the photoanode is shown in Fig. 2c. The length of ZnO nanowire is about 8 μm. There is no visible dividing line between the nanowires and the substrate in this work. The nanowires and the substrate are perfectly matched as a unity. The area marked with red dotted lines in Fig. 2c clearly reveals such a state of “no interface” between the nanowires and the substrate. However, in the FTO-based photoanode reported before, numerous grain boundary and cracks around the interface between ZnO nanowires and the FTO substrate can be found^10^,^11^,^13^,^17^. We believe the superior quality of the interface and the ZnO nanowires on GZOSC substrate is attributed to the lattice match between the nanowire and the substrate.

Low-resolution TEM image of two individual ZnO nanowires in the photoanode with a diameter of ~170 nm is presented in Fig. 2d–e. The corresponding SAED pattern plotted in the upper left inset of Fig. 2d can be indexed to the wurtzite structure of hexagonal ZnO, suggesting its growth direction along (0001)-orientation with single-crystalline nature. The results are in good agreement with the XRD analysis. The typical HRTEM image, taken from the area marked with the red frame in Fig. 2d, is illustrated in Fig. 2e. The crystal lattice fringes are clearly detected and the average distance between the adjacent lattice planes is about 0.26 nm, corresponding well to the interplanar distance of the (0001) crystal planes of the wurtzite ZnO. No crystal dislocation or defect is found from the HRTEM image, indicating a high crystalline quality of the nanowires.

**Figure 3:**
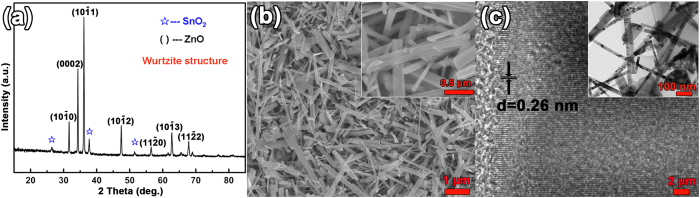
(**a**) XRD spectrum of the ZnO nanowire array/FTO photoanode. (**b**) Top view SEM image of the photoanode (the upper right inset gives its enlarged image). (**c**) HRTEM image of the ZnO nanowire taken from FTO based photoanode (Low-resolution TEM image was shown in the inset).

For comparison, DSSCs using ZnO nanowires/FTO photoanode has also been fabricated by growing ZnO nanowires on FTO substrates in the same growth conditions. The transmittance spectrum and electrical property of GZOSC and FTO conductive substrate are illustrated in the supplementary information. The electrical and optical properties suggest that GZOSC is very suitable as a transparent collector electrode. The XRD pattern of the ZnO nanowires/FTO photoanode displays many peaks which can be indexed to pure wurtzite structure of ZnO (JCPDS card: 79–0207), except those from the FTO substrate labeled with star (see Fig. 3a). The peaks in the XRD pattern demonstrate that the ZnO nanowires are grown disorderly on the FTO substrate. Top view SEM image verifies the results from the XRD pattern, and shows that the morphology of the ZnO nanowires on FTO is irregular (see Fig. 3b). ZnO nanowires on FTO are also demonstrated to have inferior crystallinity compared to those grown on single craystal GZO through the detailed examination by TEM (see Fig. 3c). From the characterization of XRD, SEM and TEM, the ZnO nanowires grown on GZOSC are demonstrated to have better crystallinity compared to those grown on FTO.

**Figure 4:**
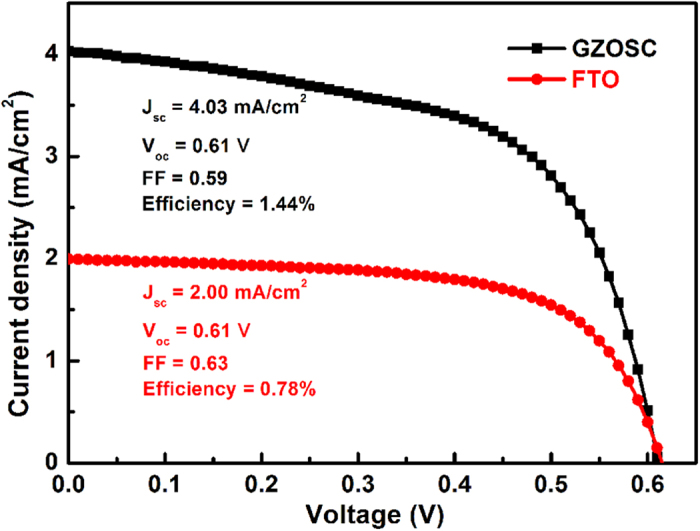
Photocurrent density–voltage curves of DSSCs with different photoanodes. The measurement was performed under 1 sun illumination.

*I*–*V* curves related to GZOSC-based DSSCs and FTO-based DSSCs are plotted in Fig. 4 and the parameters are listed in Table 1, indicating that the performance of GZOSC-based DSSCs is much better than that of FTO-based DSSCs. The photocurrent density of GZOSC-based DSSCs is close to twice of the FTO-based DSSCs. The better performance of the GZOSC-based DSSCs might be due to the high quality of the ZnO nanowire arrays and the interface between the nanowires and the substrate.

**Figure 5:**
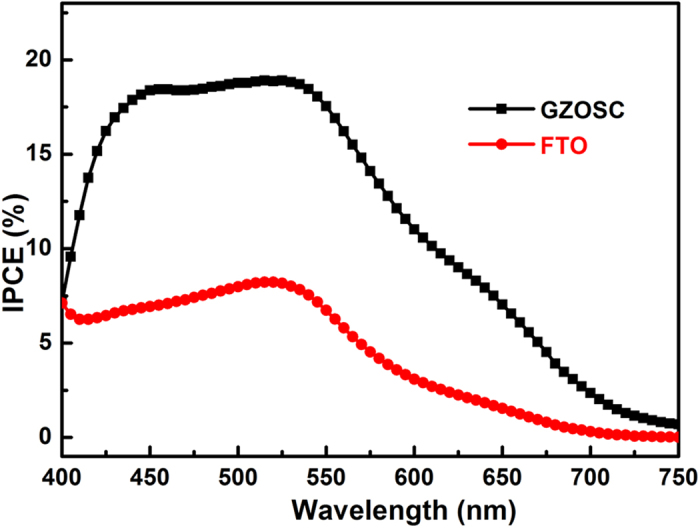
IPCE spectra for the DSSCs with different photoanode.

The incident-photon-to-current conversion efficiency (IPCE), defined as the number of electrons generated by light in the external circuit divided by the number of incident photons, is plotted as a function of excitation wavelength in Fig. 5. From the IPCE spectra of different DSSCs, it can be observed that the GZOSC-based DSSCs exhibit a photo-response over the wavelength range of 400–750 nm with a maximum value at 520 nm. Obviously, the IPCE value of GZOSC-based DSSCs (19.0%) is much higher than that of FTO-based DSSCs (8.2%) in the wavelength range of 400–750 nm. The higher IPCE implies that the sensitized GZOSC-based photoanode is more efficient than sensitized FTO-based photoanode in transmitting and/or collecting photo-excited electrons. This trend was in agreement with the variation of *J*_*sc*_ and *η* (see in Table 1)

**Figure 6:**
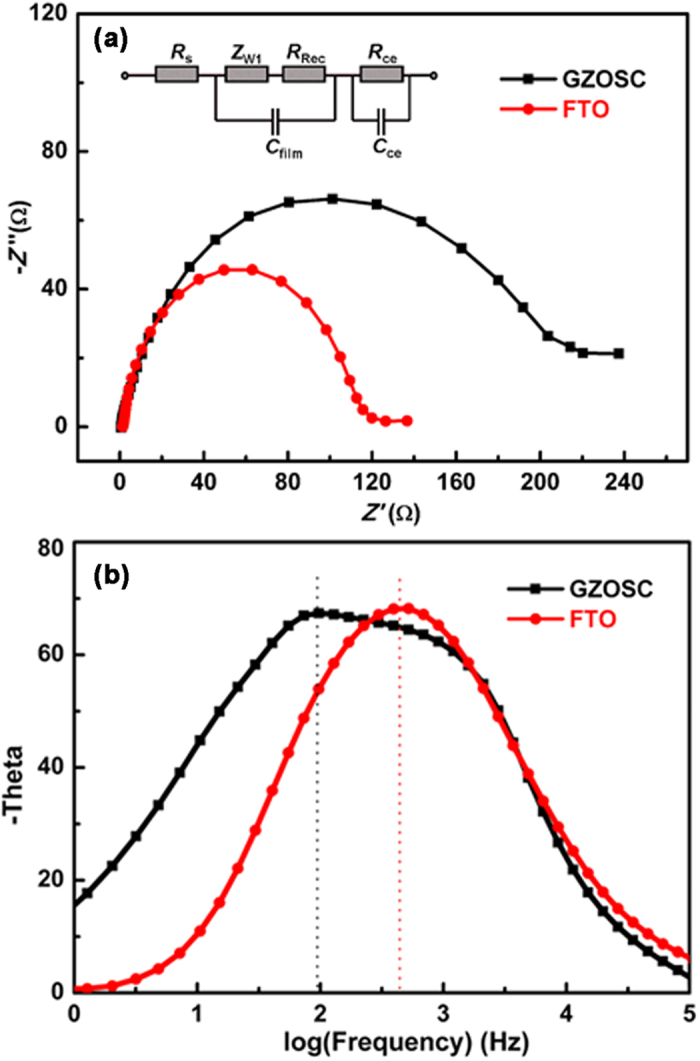
(**a**) Nyquist and (**b**) Bode phase plots of cells based on GZOSC and FTO photoanodes. Inset: the equivalent circuit of the DSSCs.

To investigate the differences in the electron-transport with different photoanodes and the interfacial charge recombination of the DSSCs, electrochemical impedance spectra (EIS) of two cells made of different photoanodes were measured. Figure 6a shows Nyquist plots of the impedance data for the cells based GZOSC and FTO in the dark by applying 5 mV AC-signal amplitude with an applied bias voltage of 0.6 V. The inset is the equivalent circuit^25^,^26^,^27^. There is one semicircle in the frequency range of 50 mHz to 1 MHz. As shown in Fig. 6, the series resistance (*R*s) for GZOSC and FTO shows no obvious difference but the fitting values of *R*s(GZO) and *R*s(FTO) are 0.57 Ω and 1.67 Ω, respectively. The fitting impedance for the charge recombination *R*_Rec_ of GZOSC-based DSSCs is 168.7 Ω, much larger than the value of FTO-based DSSCs (80.8 Ω), indicating less interfacial charge recombination within the GZOSC-based DSSCs, in which the injected electrons are extracted more effectively. It is known that the frequency is related to the electron lifetime (*τ*_n_), which can be estimated by using the relation:


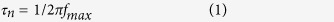


where *f*_max_ is the value at which the low frequency peak in the bode plot^28^. As can be seen in Fig. 6b, the *f*_max_ values of GZOSC and FTO are 97.2 and 521.2 Hz, and the electron lifetime values are estimated to be 1.6 ms and 0.3 ms, respectively. The longer electron lifetime in DSSCs based on GZOSC means less electron recombination for the perfect interface between the ZnO nanowire and collector electrode. The calculated result of electron lifetime indicates the perfect interface benefits the electronic transport. In addition, the previous work has demonstrated the homogeneous interface is beneficial to electrical transmission and collection efficiencies^29^,^30^. Therefore, the larger efficiency of the cell could be ascribed to the superior interfacial structure in the high quality GZOSC-based photoanode.

## Discussion

In conclusion, a novel ZnO nanowires DSSCs based on GZOSC replacing traditional FTO glass is presented in this work, which has high quality ZnO nanowires, high mobility of collector electrode and a homogeneous interface between the GZOSC and the nanowires. The efficiency of GZOSC-based DSSC was characterized by *V*_oc_ = 0.61 V, *J*_sc_ = 4.03 mA/cm^2^, *FF* = 0.59 and efficiency *η* = 1.44%. Analysis of *I*–*V*, IPCE and EIS curves demonstrated that the performance of GZOSC-based DSSCs was superior to that of FTO-based DSSCs under the same growth conditions. It is believed to be a promising way by adopting GZOSC-based photoanode to improve the performance of DSSCs.

## Methods

### ZnO nanowire arrays growth

The ZnO nanowire was grown by a CVD route. All reagents were of analytical grade and were used without further purification. A mixture of ZnO and carbon powders (1:1 wt.%) was used as source material. Argon gas with a flow rate of 200 sccm was supplied as the carrier gas in the quartz tube during heating. A quartz boat with the source material was placed at the high temperature region of the quartz reactor. The GZO substrate was placed in a position where the temperature was 450 ^o^C. GZOSC substrates were grown by the hydrothermal method and were 10 × 10 × 0.3 mm^3^ in size. Before being sent to the reactor, they were chemo-mechanically polished followed by thermal annealing in O_2_ atmosphere^22^. As soon as the temperature of the source material reached 1000 ^o^C, 10 sccm of O_2_ gas was supplied and kept for 120 min to grow the nanowire. The working pressure was maintained at 480 Pa by adjusting the orifice to the pumping line. The growth area of nanowire was controlled to be 0.25 cm^2^ by using a mask during the growth. The overall process was performed without using any catalyst.

### Solar cell assembly and photovoltaic measurement

For the sensitization of the ZnO nanowire, the ZnO nanowire was immersed into a 0.3 mM solution of the dye (N719:D131 = 1:1) in ethanol for 2 h. The dye-sensitized photoanode with an active area of 0.25 cm^2^ was incorporated into a sandwiched solar cell. Pt was sputtered on FTO glass as counter electrode. The electrolyte consisted of 0.6 M 1,2-dimethyl-3-n-propylimidazolium iodide, 0.1 M LiI, and 0.05 M I_2_ in acetonitrile with 0.5 M 4-tertbutylpyridine.

The photovoltaic performance of the solar cells was measured with a source meter (Keithley 2400). An AM1.5 solar simulator PEC-L11 (Peccell Technology Co. Ltd., with a 1000 W Xe lamp and an AM1.5 filter) was used as the light source (100 mW cm^−2^). Action spectra of the IPCE were collected by PEC-S20 (Peccell Technology Co. Ltd.). The incident light intensity was calibrated with a standard solar cell for amorphous silicon solar cell produced by Japan Quality Assurance Organization. EIS of GZOSC-based electrodes were measured by using a ZAHNER (IM-6).

### Characterization

Hall effect was tested in the Van der Pauw configuration by Lake Shore 7700A Hall effect measurement system to detect the electric properties of the conductive substrate. X-ray diffraction (XRD) was used to identify the crystal phase of the ZnO nanowire. Diffraction data were recorded by using a PANalytical X’Pert PRO diffractometer with Cu Kα radiation (40 kV, 40 mA) in continuous scanning mode. The morphology of ZnO nanowire was characterized by field-emission scanning microscopy (SEM, JSM-6700F, JEOL, Tokyo, Japan). Transmission electron microscopy (TEM) and selected area electron diffraction (SAED) pattern images were taken by using a TECNAI F20 at 200 kV.

## Additional Information

**How to cite this article**: Hu, Q. *et al.* ZnO nanowires array grown on Ga-doped ZnO single crystal for dye-sensitized solar cells. *Sci. Rep.* **5**, 11499; doi: 10.1038/srep11499 (2015).

## Supplementary Material

Supplementary Information

## Figures and Tables

**Figure 1:**
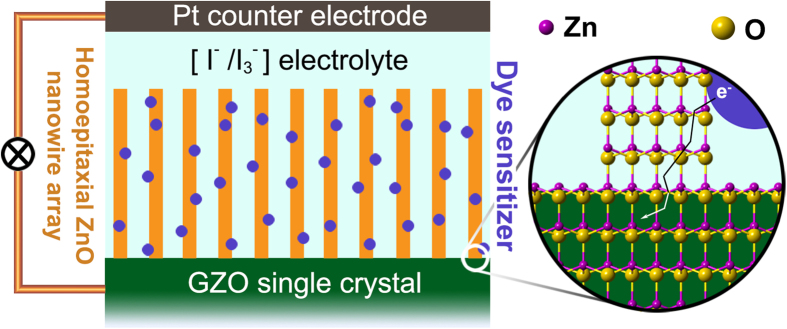
The schematic diagram of the GZOSC-based DSSCs (Left) and the injected electrons transport smoothly in homogeneous interface (Right).

**Table 1:** *I*–*V* parameters of DSSCs with different photoanodes under 1 sun illumination.

photoanode	*J*_sc_ (mA cm^−2^)	*V*_oc_ (V)	*FF*	*η* (%)
GZO single crystal	4.03	0.61	0.59	1.44
FTO	2.00	0.61	0.63	0.78
