# Supplementary material for: ZnO nanowires array grown on Ga-doped ZnO single crystal for dye-sensitized solar cells
Source: Sci Rep. 2015 Jun 23;5:11499. doi: 10.1038/srep11499 (PMC4477229; doi:10.1038/srep11499)
Supplement: Supplementary Information [file srep11499-s1.doc]

- **Supplementary Information**

**ZnO nanowires array grown on Ga-doped ZnO single crystal for dye-sensitized solar cells**

Qichang Hu^1^, Yafeng Li^2^, Feng Huang*^1^, Zhaojun Zhang^1^, Kai Ding^1^ Zhang Lin^1^,and Mingdeng Wei*^2^

1. Key Laboratory of Optoelectronic Materials Chemistry and Physics, Fujian Institute of Research on the Structure of Matter, Chinese Academy of Sciences, Fuzhou, Fujian, 350002, People's Republic of China

2. Institute of Advanced Energy Materials, Fuzhou University, Fuzhou, Fujian 350002, People's Republic of China

Correspondence to [fhuang@fjirsm.ac.cn] and [wei-mingdeng@fzu.edu.cn]

**
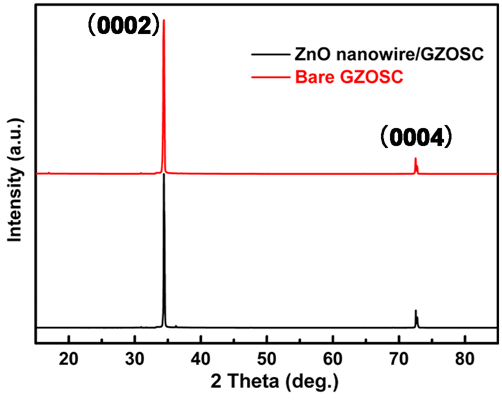
**

**Fig. S1 The XRD patterns of bare GZO and ZnO nanowire/GZOSC.**

The XRD patterns of bare GZO and ZnO nanowire/GZOSC are shown in **Figure S1.** They have two diffraction peaks at 34.4° and 72.4°, which can be indexed as (0002) and (0004) wurtzite structure of ZnO, respectively. The same diffraction angle of the peak shows that they have the same lattice parameters.


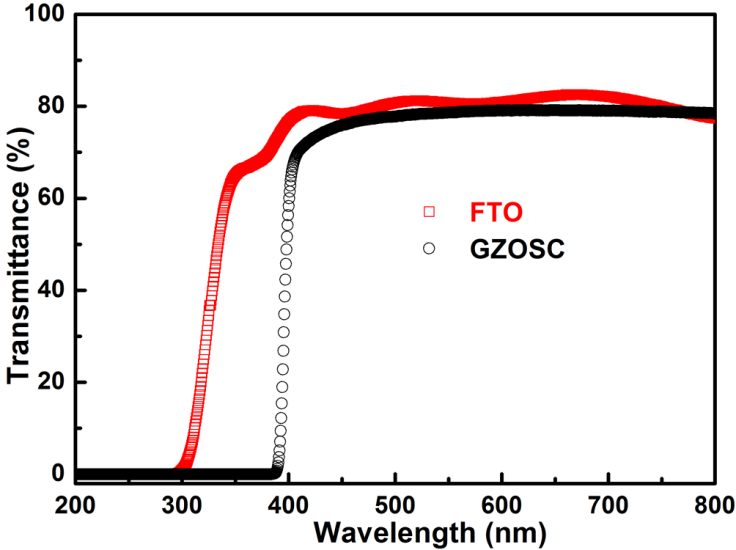


**Fig. S2 Transmittance spectra for FTO conductive substrate and** **GZOSC (□: FTO conductive substrate;○: GZOSC )**

Table S1 Electronic properties of the FTO conductive glass and GZO single crystal

| Sample | Resistance(Ω·square^-1^) | Mobility(cm^2^·V^-1^·S^-1^) | carrier concentration(cm^-2^) |
| --- | --- | --- | --- |
| FTO | 11.84 | 25.3 | -2.086×10^16^ |
| GZO | 0.3061 | 137.2 | -1.490×10^17^ |

It can be seen from Figure S2 that GZOSC (78.5%) and FTO (around 80%) have similar transmittance in the visible range (400 ~800nm). Hall measurement results summarized from Table S1 indicate that GZOSC has a lower sheet resistance, higher electron mobility than FTO conductive glass. The high conductivity and mobility is beneficial to the transport of injected electrons. The high conductivity and mobility is beneficial to the transport of injected electrons. These electrical and optical properties suggest that GZOSC is very suitable as a transparent collector electrode.
